# The influence of early life exposures on the infant gut virome

**DOI:** 10.1080/19490976.2025.2501194

**Published:** 2025-05-21

**Authors:** Yichang Zhang, Josué L. Castro-Mejía, Ling Deng, Shiraz A. Shah, Jonathan Thorsen, Cristina Leal Rodríguez, Leon E. Jessen, Moïra B. Dion, Bo Chawes, Klaus Bønnelykke, Søren J. Sørensen, Hans Bisgaard, Sylvain Moineau, Marie-Agnès Petit, Jakob Stokholm, Dennis S. Nielsen

**Affiliations:** a Department of Food Science, University of Copenhagen, Frederiksberg, Denmark; b Copenhagen Prospective Studies on Asthma in Childhood, Copenhagen University Hospital, Herlev-Gentofte, Gentofte, Denmark; c Novo Nordisk Foundation Center for Basic Metabolic Research, Faculty of Health and Medical Sciences, University of Copenhagen, Copenhagen, Denmark; d Department of Health Technology, Section for Bioinformatics, Technical University of Denmark, DTU, Lyngby, Denmark; e Département de Biochimie, de Microbiologie, et de Bio-Informatique, Faculté des Sciences et de Génie, Université Laval, Québec, QC, Canada; f Groupe de Recherche en Écologie Buccale, Faculté de Médecine Dentaire, Université Laval, Québec, QC, Canada; g Department of Biology, University of Copenhagen, Frederiksberg, Denmark; h Félix d’Hérelle Reference Center for Bacterial Viruses, Faculté de médecine dentaire, Université Laval, Québec, QC, Canada; i Université Paris-Saclay, INRAE, AgroParis Tech, Micalis Institute, Jouy-en-Josas, France

**Keywords:** Environmental exposures, infant, gut microbiota, virus, phage, bacterial host, metabolism

## Abstract

The factors influencing the establishment of the gut bacterial community in early life are fairly well studied. However, the factors shaping the infant gut virome remain elusive. Interestingly, early life gut virome imbalances have recently been linked with increased risk of developing diseases like type 1 diabetes and asthma. We utilized the deeply phenotyped COPSAC2010 cohort to investigate how environmental factors influence the gut virome at one year age. We demonstrate that the presence of older siblings as well as residential location (urban or rural) had the strongest impact on gut virome composition at 1 year of age. A total of 16,118 species-level clustered viral representative contigs (here termed viral Operational Taxonomic Units – vOTUs) were identified and of these 2105 vOTUs varied in abundance with environmental exposures. Of these vOTUs 94.1% were phages mainly predicted to infect *Bacteroidaceae*, *Prevotellaceae*, and *Ruminococcaceae*. Strong co-abundance of phages and their bacterial hosts was confirmed underlining the predicted phage-host connections. Furthermore, we found some gut viruses affected by environmental factors encode enzymes involved in the utilization and degradation of major dietary components, potentially affecting infant health by influencing the bacterial host metabolic capacity. These findings provide a valuable insights for understanding the early life factors that predispose to autoimmune and metabolic disorders.

## Introduction

Early life gut microbiome (GM) establishment plays a fundamental role in shaping host physiology and health^1,2^ with early life GM imbalances being linked to onset and progression of chronic diseases later in life, such as obesity,^3^ diabetes,^4,5^ and asthma.^2^

To date, GM research has generally focused on understanding the importance of the bacterial GM component, but recent findings indicate that the vast and diverse population of viruses found in the gut (collectively called the “gut virome”) also play a prominent role in gut microbial ecology^6–9^. Amidst these biological entities, bacterial viruses, also termed bacteriophages (phages), are the most diverse and abundant particles of the GM^9–11^ representing a major reservoir of genetic diversity influencing not only GM composition but also the GM metabolic potential.^12,13^ Disease-specific alterations in the gut virome have been reported in several chronic conditions^14^ such as inflammatory bowel disease,^15^ colorectal cancer,^16^ necrotizing enterocolitis in preterm infants,^17^ severe acute malnutrition,^18^ type-1 diabetes^19,20^ and other autoimmune diseases such as rheumatoid arthritis.^21^ Very recently, we have demonstrated that infant gut virome imbalance in the temperate phage pool at the age of 1 year is associated with increased risk of developing asthma before school age. Interestingly, it was found that this effect was additive to the increased risk of developing asthma due to imbalanced gut bacteriome at the age of 1 year, meaning that the influence of gut virome composition on asthma is not only mediated via influencing the bacterial gut component but also independent of the bacteriome.^22^ The role of the gut virome in shaping the GM is further underlined by the observation that fecal virome transfer from healthy donors to recipients with a dysbiotic GM prevent or ameliorate symptoms associated with metabolic^23^ and gastrointestinal^24,25^ disorders.

While various early-life factors such as birth mode, siblings, diet and exposure to antibiotics have been found to influence the development of the gut bacterial community,^1,26^ little is known about which factors shape the gut virome. The few attempts that have characterized the gut virome early in life have revealed that its composition is highly dynamic,^27–29^ affected by delivery mode,^6,30^ maternal factors such as gestational diabetes^30^ and the first bacterial colonizers^31^ as well as being enriched in phages belonging to the *Microviridae* family.^10,28^ Moreover, its transmission-dynamics after birth follows a stepwise assembly, with breastfeeding playing a protective role against eukaryotic viral infections.^32,33^ Understanding how environmental exposures and phenotypes intertwine the vector space conformed by viruses, bacteria, host, and their functional attributes remains hitherto an unsolved task.

In a recent detailed investigation of the infant gut virome, we showed a massive diversity of hitherto undescribed phages.^9^ In this cross-sectional study of the gut virome of 645 infants at 1 year of age enrolled in the COPSAC_2010_ cohort^34^ more than 10000 viral species distributed over 248 viral families and 17 viral order-level clades were detected.^9^ Recently, gut virome imbalance was as mentioned above linked to increased risk of developing asthma before school age in this cohort.^22^

Early life environmental exposures have influenced gut bacteriome development early in life.^2,26,35,36^ Here we expand these findings by investigating how social, pre-, peri- and postnatal factors influence the gut virome composition at 1 year of age. Our findings demonstrate how early life exposures are linked to the abundance of specific viruses, as well as their co-abundance and concordance with their predicted bacterial hosts. Metabolic functions encoded in the genomes of these viruses displayed enrichment of genes important for bacterial physiology in response to exposures, some of which are likely associated with dietary elements (e.g., degradation of complex carbohydrates) and others that may influence infant growth and health.

## Results

### Composition of DNA viruses in the gut of Danish infants

**Figure 1.**
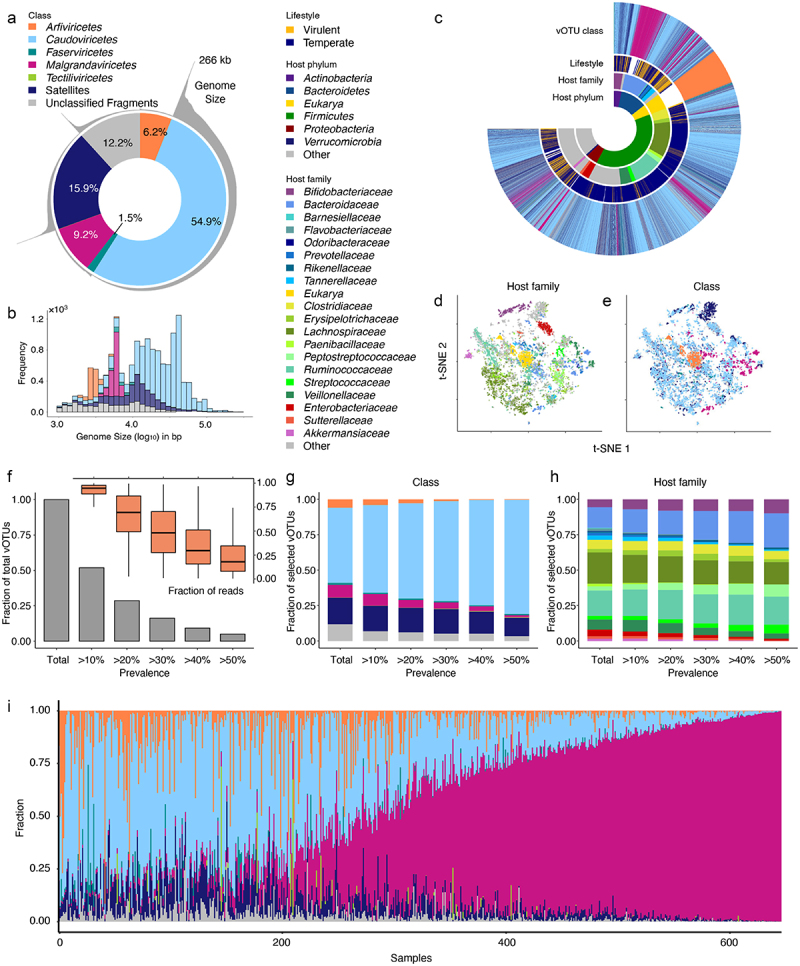
Virome structure of the infants enrolled in the COPSAC_2010_ cohort. (a) Distribution of the 16,118 vOTUs identified colored by their taxonomic class annotation. (b) Cumulative frequency of viral genomes (kb) identified by their taxonomic class annotation. (c) Circular diagram showing the distribution of vOTUs colored by their targeted bacterial hosts (at phylum and family levels), viral class and lifestyle. (d,e) *t*-Stochastic Neighbor Embedding (t-SNE) plots clustering *tetra*-mer vOTUs profiles identified according to host family (d) and viral class (e). (f–h) Percentage of vOTUs that appear at a specific prevalence (f), and vOtus’ distribution colored by their taxonomic class (g) and host family (h). (i) Relative abundance of vOTUs across all samples at the class level. Samples were sorted by *Malgrandaviricetes*abundance.

A total of 645 stool samples from 1-year-old infants in the COPSAC_2010_ cohort^34^ were obtained and analyzed.^9^ Virions were isolated, concentrated and their genome was sequenced using a shotgun metagenome strategy.^9,37^ Following assembly, a total of 16,118 species-level clustered viral representative contigs (here termed viral Operational Taxonomic Units – vOTUs) were obtained. Around 70% of the vOTUs were affiliated to five viral classes (*Arfiviricetes, Caudoviricetes, Faserviricetes, Malgrandaviricetes* and *Tectiliviricetes*) (Figure 1(a,l)). Almost 18.8% of the vOTUs (*n* = 3,029) were considered putative satellite phages as contigs lacked genes coding for structural proteins but encoded other viral proteins (e.g., integrases or replicases) and were conserved in size and gene content across multiple samples. In addition, 11.8% of the vOTUs (*n* = 1,895) were categorized as unclassified viral fragments (Figure 1(a)).

The largest genomes (>10 kb) were observed among *Caudoviricetes*, which constituted the vast majority of vOTUs (Figure 1(b)). The genomes, dominated by *Caudoviricetes* (tailed, double-stranded DNA phages) and *Malgrandaviricetes* (non-tailed, single-stranded DNA phages), followed a bi-/multi-modal distribution (Hartigans’ Dip test, *p* < 0.0001) based on their genome sizes (Figure 1(b)).

Bacterial hosts as well as lifestyle (temperate/virulent) of the vOTUs were predicted using CRISPR spacers and the presence of integrases,^9^ respectively (Figure 1(c)). Because phages tend to have comparable *k-*mer frequencies to those of their hosts,^38,39^ we also performed dimensionality reduction on tetramer vectors to confirm global host associations as a complement to our viral taxonomy.^9^ Using unsupervised stochastic neighbor embedding (t-SNE) dimensionality reduction, vOTUs targeting the same hosts as determined by CRISPR spacers (Figure 1(d)) or belonging to the same viral classes (Figure 1(e)) were found to clearly cluster together. Previously only *Enterobacteriaceae* and *Bacteroidetes* have been shown to be the hosts of non-tailed *Malgrandaviricetes*,^40^ but when examining the bacterial hosts, we observed that in addition to *Bacteroidetes*, also *Ruminococcaceae, Clostridiaceae, Erysipelo-trichaceae* and *Sutterellaceae* are predicted as hosts of *Malgrandaviridetes* viruses (Figure 1(c) and S1). With respect to lifestyle, vOTUs predicted to infect *Streptococcaceae* and most families of *Bacteroidetes* exhibit a higher proportion of virulent phages compared vOTUs predicted to infect other families (Figure 1(c) and Table S2).

The distribution of vOTUs was very individual-specific, with less than 5% of vOTUs appearing in more than 50% of the samples (Figure 1(f)). However, this still adds up to around 800 vOTUs that are shared among a majority of infants and representing, on average, more than 20% of the sequencing reads (Figure 1(f)). The proportion of vOTUs classified as *Caudoviricetes* (Figure 1(g)) as well as those infecting *Bacteroidaceae* and *Bifidobacteriaceae* (Figure 1(h)) increased as a function of prevalence.

### Environmental exposures influence viral diversity

**Figure 2.**
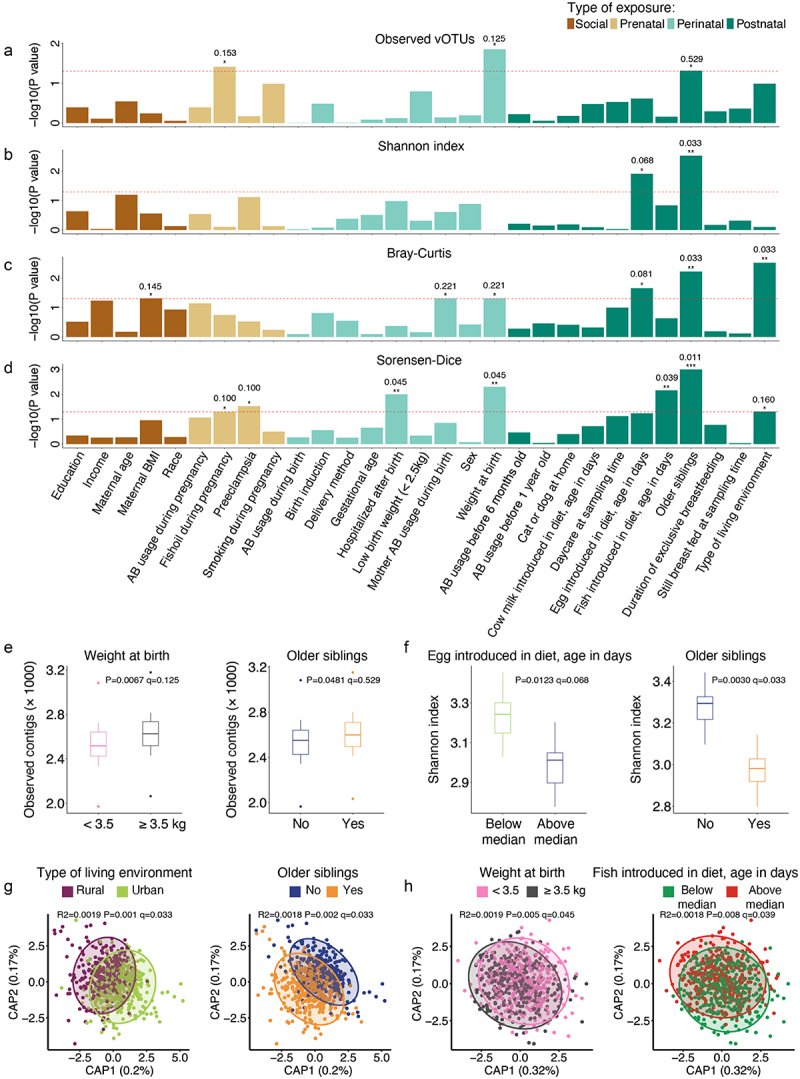
Virome diversity and composition covariates with early life exposures. (a–d) Barplot showing the strength of associations (-log10 p-value) of the alpha diversity metrics observed vOTUs (a) and Shannon index (b) across different exposures (linear mixed model) as well as beta diversity using distance-based redundancy analysis (db-RDA) on bray-curtis dissimilarity (c) and sorensen-dice distance (d) matrices. Statistical significance levels are represented as *p* < 0.001 (***), *p* < 0.01 (**) and *p* ≤ 0.05 (*). P-values were adjusted by Benjamini – Hochberg method and shown as q-values (only shown for significant exposures). (e,f) distribution of observed vOTUs for weight at birth and siblings (e) and Shannon index for siblings and dietary introduction of egg (f). Dietary introduction of egg is indicated in days. (g,h) db-RDA constrained-components based on bray-curtis distances for location and siblings (g), and sorensen-dice distances for weight at birth and dietary introduction of fish (h).

A range of pre-, peri-, and postnatal as well as social factors were recorded for the enrolled infants and their families (Supplementary Table S1). Having older siblings was associated with higher vOTU richness (linear mixed model, *p* = 0.048, q = 0.529, estimate = 69.14, 95% CI = [0.58, 137.52]) and lower evenness (Shannon *H’*) (linear mixed model, *p* = 0.003, q = 0.033, estimate = −0.30, 95% CI = [−0.50, −0.10]) (Figures 2(a–b, e–f)) at 1 year of age. Likewise, a higher birth weight was linked to higher vOTU richness (linear mixed model, *p* = 0.007, q = 0.125, estimate = −85.76, 95% CI = [−153.98, −17.56]) (Figure 2(a,e)). Dietary factors were also found to influence the gut virome at 1 year of age, with late introduction of eggs in the diet being associated with lower viral evenness (Shannon *H’*) (linear mixed model, *p* = 0.012, q = 0.068, estimate = 0.25, 95% CI = [0.05, 0.45]) (Figure 2(a,f)). The mothers were enrolled in a nested randomized placebo-controlled trial of fish oil to the mothers during the third trimester of pregnancy.^41,42^ Receiving fish oil during pregnancy was associated with increased gut vOTU richness (linear mixed model, *p* = 0.038, q = 0.153, estimate = 71.60, 95% CI = [3.90, 139.22]) of the infants at 1 year of age (Figure 2(a)). The design also examined the difference in vitamin D between high and standard doses,^43^ which had no effect on the viral community in our analysis. Interestingly, other factors that have been found to influence the bacterial GM component during infancy such as birth mode, use of antibiotics, and duration of exclusive breastfeeding^1,26^ did not seem to influence gut virome alpha-diversity measures at 1 year of age in this cohort (Figure 2(a–b)).

Regarding virome composition, Bray-Curtis dissimilarity analysis (weighted measure, which is therefore mainly influenced by more abundant vOTUs) indicate a link (ANOVA-like permutation test, *p* = 0.049, q = 0.145, R2 = 0.0016) between maternal body mass index (BMI) and virome composition at 1 year of age (Figure 2(c) and S2A); while Sørensen-Dice distance (unweighted binary metric and therefore mainly influenced by more rare vOTUs) revealed that a number of pre- and perinatal exposures were linked with virome composition differences (ANOVA-like permutation test, *p* ≤0.05), namely weight at birth, fish oil supplementation during pregnancy, hospitalization after birth, and preeclampsia (Figure 2(d,h) and S2B). Both Bray-Curtis and Sørensen-Dice metrics showed significant differences in virome composition for children having older siblings (ANOVA-like permutation test, *p* = 0.006, q = 0.033, R2 = 0.0018 and *p* = 0.001, q = 0.011, R2 = 0.0029 for Bray-Curtis and Sorensen-Dice dissimilarity metrics, respectively), and whether the family was living in an urban or a rural area (ANOVA-like permutation test, *p* = 0.003, q = 0.033, R2 = 0.0019 and *p* = 0.049, FDR = 0.160, R2 = 0.0016 for Bray-Curtis and Sorensen-Dice dissimilarity metrics, respectively) (Figure 2(c–d,h) and S2A-D).

### Environmental exposure variables influence the abundance of specific vira

**Figure 3.**
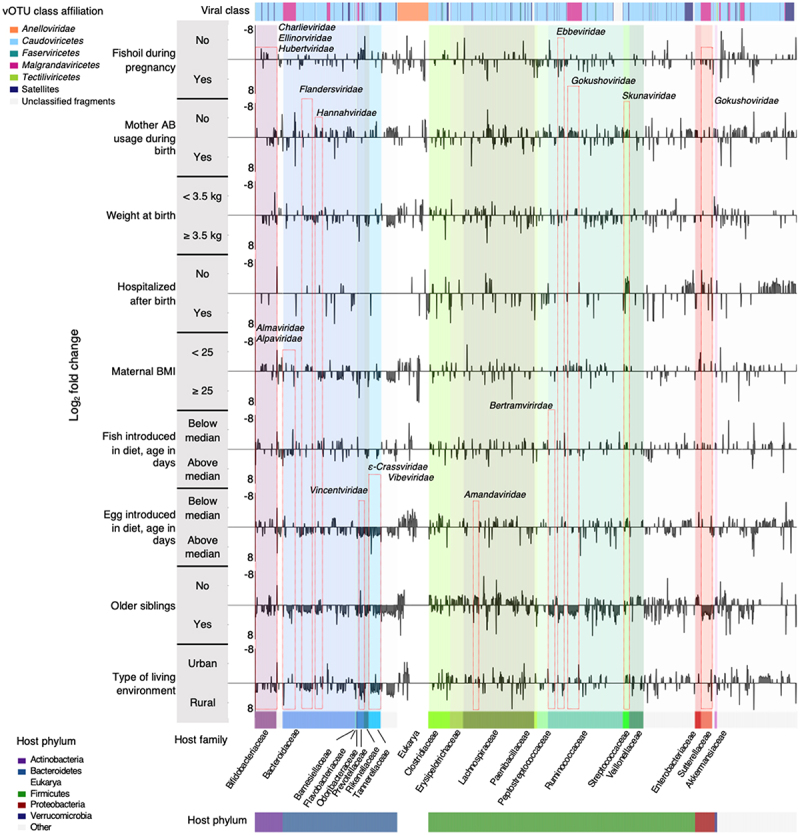
Viral host family, relative abundance and lifestyle associate with environmental exposures at one year of age. Visualization of differential abundance analysis of 2105 vOTUs across the nine exposures significantly associated with virome diversity and composition. Log_2_ Fold change panel displays the change in abundance between the two groups for each exposure. The viral families to which vOTU belongs, surrounded by red boxes, are labeled. Adjusted *p* ≤ 0.001 and Log_2_-Fold changes ≥ |1| were used to select differentially abundant vOTUs.

Subsequently, we determined how the distribution of vOTUs differed between the nine exposures (Figure 2c–d) found to significantly influence overall gut virome composition (preeclampsia was not included due to highly unbalanced sample size, see Supplementary Table S1). A total of 2,105 differentially abundant vOTUs affiliated to 173 viral families and 19 families of bacterial hosts were identified by DESeq2, with having older siblings being associated with 822 differential abundant vOTUs, while being hospitalized after birth being associated with 212 differential abundant vOTUs (Figure 3). For perinatal covariates, vOTUs differing in abundance were predicted to infect a range of different hosts, but interestingly revealed a pronounced lower abundance of vOTUs predicted as having *Bacteroidaceae*, *Ruminococcaceae* and *Streptococcaceae* as hosts with maternal antibiotic usage and hospitalization after birth (Figure 3). Postnatal factors like specific dietary patterns (late introduction of eggs in the diet), presence of older siblings in the house and living in a rural environment, were associated with a higher abundance of vOTUs infecting *Bifidobacteriaceae*, *Bacteroidaceae*, *Prevotellaceae*, *Tannerellaceae*, *Ruminococcaceae* and *Sutterellaceae*.

To further integrate these findings in the context of the gut bacterial component, we used 16S rRNA gene (V4 region) amplicon sequencing (bacterial OTUs – bOTUs) data previously published for this cohort^2^ to determine virus-host co-abundances. Spearman correlation coefficients (ρ) were calculated between the abundance of the above identified differentially different abundant vOTUs and bOTUs across samples. Only bOTUs that were strongly associated (ρ≥0.3 and p-values ≤0.05) with at least one vOTU were retained. In most cases, when a vOTU was correlated with a bOTU, the bOTU family aligned with the predicted host family of the vOTU. However, some exceptions were observed, such as vOTU infecting *Clostridiaceae* and *Lactobacillaceae* bOTU, reflecting the complexity of phage-host dynamics in the gut microbiome (Figure S3A). These virus-host co-abundances indicate there is a high degree of inter-relatedness between phages and their host in response to environmental exposures. This was supported by the fact that the same perinatal and postnatal covariates were also significantly associated with bOTU diversity and composition (Figure S4A-D). Overall, the 91 co-abundant vOTUs (ρ ≥ 0.3 and p-values ≤0.05), vOTUs that infect the same bacterial host family were in most cases closely related genetically, indicating a high degree of co-evolution between bacterial hosts and the phages that infect them (Figure S3B). To confirm the above-mentioned findings, we repeated the analysis of virus-host co-abundances using shotgun metagenomic data from the same cohort.^44^ We found again that viruses and their bacterial hosts were highly correlated supporting the same conclusion as above (Figure S4E).

Because of the close relationship between phages and their bacterial hosts, we sought to determine whether bacterial communities mediate the effect of environmental exposures on the phages. Through mediation analysis, our findings indicate that, for certain exposures – such as having older siblings – bacterial communities partially mediate their influence on phage composition (Figure S5A). In contrast, other factors, including living environment and birth weight, operate more direct effects on the phageome (Figure S5B-C). Finally, some exposures such as age for introduction of eggs in the diet did not exhibit significant direct or indirect effects (Figure S5D).

### Functional profiles of gut viruses are linked with environmental exposures

Differentially abundant vOTUs were subjected to gene (open reading frame, ORF) prediction, and annotated based on KEGG Orthology (KO) using KofamScan.^45^ As seen from figure S6A, 0.82% of genes matched known metabolism-related orthologs, while the remaining genes with KO assignments (8.48% of predicted genes) encoded genes related to genetic information processing and signaling and cellular processes, representing typical viral-associated traits required to accomplish replication.^46^ The remaining 90.7% of the predicted genes were not annotated by the database.

**Figure 4.**
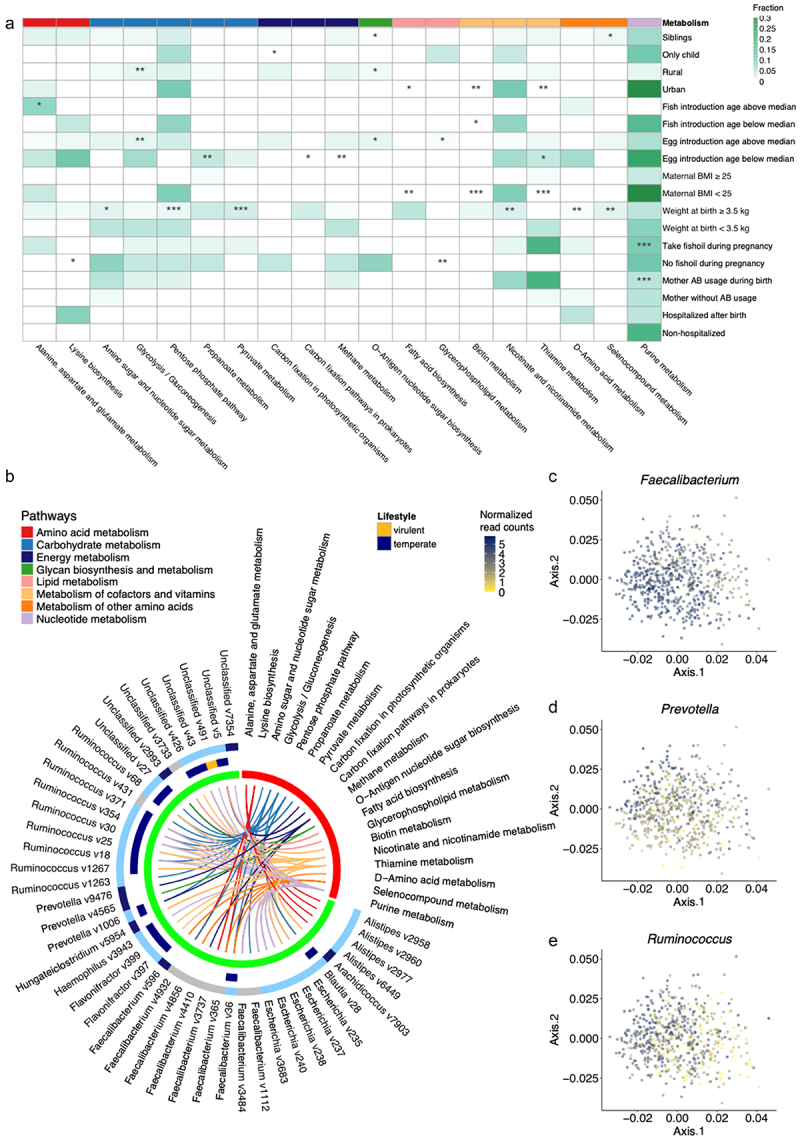
Abundance of phage accessory genes differ in connection with exposures. (a) Abundance of genes (3^rd^ level KEGG pathway) in the virome of infants with significant (*p* ≤ 0.05) enzymatic enrichments that are associated with the presence of siblings and residential location. (b) Viral host families that contribute to metabolism pathways. the outermost circle represents the vOTU class. The second circle represents the viral lifestyle (virulent vs. temperate). In the innermost circle, red represents metabolic pathways and green represents the vOtus. The pathway and the family of the vOTU host are labeled. (c–e) Bacterial points extracted from the procrustes analysis (e). The points are colored according to the abundance of the specific genus in each sample.

Next, we focused on determining genes with metabolic functions having the potential to enhance host fitness and drive metabolic reprogramming of the bacterial host.^47^ The gut virome of infants with older siblings were enriched in genes related to O−antigen nucleotide sugar biosynthesis and seleno-compound metabolism, while infants without siblings were enriched in genes related to carbon fixation in photosynthetic organisms (Fisher’s exact test, *p* < 0.05; Figure 4(a)) (the link to photosynthetic microorganisms may be caused by the KEGG database not being optimized for vira). The gut of infants living in rural areas or that were introduced to eggs in their diet later in life (above the median age when eggs were introduced in the diet) were enriched in viral encoded genes associated with glycolysis/gluconeogenesis and O−antigen nucleotide sugar biosynthesis, whereas the gut of infants living in urban areas or that were introduced to eggs relatively early in life were enriched in viral genes associated with thiamine metabolism. Infants with birth weight above the median or whose mothers who had a pre-pregnancy BMI < 25 also encoded genes involved in diverse pathways involved in, e.g., vitamin synthesis. Further, the gut virome of infants whose mothers received fish oil during pregnancy or were prescribed antibiotics during delivery encoded genes related to purine metabolism (Figure 4(a)).

To determine how virally encoded gene functions associate with the microbial composition, we linked back enriched genes to the vOTU of origin (Figure 4(b)). 94% of lifestyle predicted vOTUs (*n* = 17) were temperate. Genes associated with two classes of amino acid metabolisms (i.e. alanine, aspartate and glutamate metabolism and lysine biosynthesis) were conserved across *Alistipes* and *Faecalibacterium* targeting vOTUs, respectively. In addition, multiple carbohydrate metabolism enzyme encoding genes were found to be widely encoded by *Blautia*, *Prevotella*, *Ruminococcus* and *Faecalibacterium* targeting vOTUs. These encoded enzymes including L−lactate dehydrogenase, ribose−phosphate pyrophosphokinase and aldose 1−epimerase (Figure S6D). Energy metabolism genes were found in *Prevotella and Faecalibacterium* targeting vOTUs, while nicotinate and nicotinamide metabolism genes were mapped in *Ruminococcus* and *Escherichia* targeting vOTUs (Figure 4(b)).

Phage-host co-abundance (Figure S3A), was further confirmed by Procrustes analysis. The linking of the virome and bacteriome compositions revealed a strong correlation one to another (*p* < 0.001, *r* = 0.52) (Figure S6B-C). The cumulative abundance of all bOTUs belonging to the bacterial genera *Ruminococcus, Prevotella* and *Faecalibacterium*, which were found to be the main bacterial host of vOTUs carrying the above metabolic genes (Figure 4(b)) and having previously been reported to be highly associated with stable viral communities,^48^ was highly correlated with rural vs. urban living and having older siblings (Figure 4(c–e)). These results emphasize the potential role of phage-host association in metabolic regulation.

## Discussion

The gut of healthy newborns is usually devoid of viruses at birth, but it is rapidly colonized afterward.^28,32^ Still relatively few studies have focused on the assembly of the gut virome within the first year of life and the factors that influence it^6,27,28,32^ and even less is known about the environmental exposures that shape the gut virome.

Here, we leveraged a massive gut virome dataset from healthy infants at 1-year of age, and integrated measures of viral diversity such as sequence composition, viral hosts, and phage lifestyles,^9^ (see Figure 1) with social, pre-, peri- and postnatal environmental exposures. We revealed the effects of these exposures on viral community and the possible effects on metabolism.

In previous reports, *Crassvirales* (class *Caudoviricetes*) and *Microviridae* (class *Malgrandaviricetes*) phages were found to be the two most abundant viral groups in the adult human gut, with their relative abundance being negatively correlated.^19,48–51^ Here, in one-year-old infants, a similar observation was made, members of the *Caudoviricetes* and *Malgrandaviricetes* classes were the most abundant phages.

Interestingly, ongoing exposures such as having older siblings and residential location, as well as past exposures (e.g., birth weight, preeclampsia) were linked with gut virome composition at one year of age. However, it is still possible that the prenatal and perinatal exposures still influenced the immune education earlier in life and remnants of the interplay are still tangible at 1-year of age.^5^ In line with this, previous studies have shown that delivery mode and gestational diabetes influence the infant gut virome composition in the period immediately after birth and with gestational diabetes even being associated with higher abundance of pathogenic herpesvirus and poxvirus in meconium samples.^30,52^ Among the exposures significantly influencing the gut virome composition, the largest effect sizes were from residential location (rural vs. urban) and having older siblings (see Figure 2(c,d)). Interestingly, urbanization has been reported to have a significant impact on the composition of the adult viral community, with individuals living in urban areas having higher abundance of *Lactococcus* (family *Streptococcaceae*) phages.^53^ The latter is presumably associated with the consumption of dairy products. We show that the living environment also affects the gut virome of infants, and that *Streptococcaceae* targeting phages are also more abundant in infants living in urban areas, possibly reflecting differences in dietary habits rather than residence *per se* (Figure 3).

Having older siblings influences the development of the bacterial community in early life^35,54,55^ and here we show that having older siblings is also associated with gut virome composition at 1 year of age. Both low as well as high birth weight has been associated with increased asthma risk during childhood.^56,57^ Interestingly, in the present study, we also find that infants with a birth weight <2.5 kg differ in gut virome composition at age 1 year, relative to infants born with a weight >2.5 kg. Importantly, focusing on the curated DNA phage community (i.e. not the total vOTU pool, but only the fraction predicted as being bacteriophages) we recently demonstrated that phageome imbalances are associated with increased risk of developing before school age, but also that having older siblings was negatively associated with higher virome asthma signature score, implying a lower asthma rate, showing how the influence of environmental exposures on virome composition also have implications for child health.^22^ Importantly, from a translational angle, early-life exposures may affect the establishment of health phenotypes, such as the protective role of breastfeeding against eukaryotic-viral infections in the neonatal period.^32^ Combining gut bacterial compositional data with gut virome composition (Figure S3A and S6B-C) in our cohort elucidates the co-abundance of phages and their hosts, underlying the role of phage-host interactions in shaping the GM. Most of these viruses (Figure S3A) have temperate lifestyles, as evidenced by the presence of genes coding for integrases. Thus, these temperate phages appear to have the ability to integrate their genome into the bacterial hosts and become prophages at some point. In a recent study^22^ investigating the influence of early-life gut virome imbalances on the risk of developing asthma before school-age we used mediation analysis to show that gut viruses influence asthma partly mediated via gut bacteria partly independent of gut bacteria. Using a similar approach we in the present study show that for some exposures like the having older siblings, the influence on the gut phageome is partly mediated via gut bacteria. However, for other exposures such birth weight and living environment, the influence on the gut phageome is largely mediated independently of gut bacteria. How phages can influence the human host without being mediated via bacteria is far from fully understood but interactions with immune system via, e.g., TLR9 receptors likely play a role.^22,58^

Gut virome members have the potential to modulate biochemical processes.^12,13,59^ The functional prediction of the genes derived from vOTUs co-varying with exposures, revealed up to 90% of genes with unknown functions. It emphasizes that proteins with yet uncharacterized functions are potentially playing a role in the regulation of human host phenotypes. Certain predicted gene functions linked to metabolic activities, such as alanine, aspartate and glutamate metabolism, amino sugar and nucleotide sugar metabolism and glycolysis/gluconeogenesis, which are likely associated with dietary intake and degradation of macronutrients, were associated with fish in the diet, birth weight, residence location and egg in the diet (Figure 4(a)). Birth weight also show negative correlation with higher virome asthma signature score (asthma rate),^22^ and time-to-asthma analysis proved that the effects of phages on asthma were additive and statistically independent of the bacterial gut microbiome component.^22^ Maternal obesity alters fatty acid metabolism and changes in gene expression of lipid metabolism in infants, which cause a higher risk of developing obesity and its complications, neuropsychiatric disorders and asthma.^60,61^ We find here that viral genes associated with normal weight mothers were predominantly enriched in fatty acid biosynthesis compared to obese mothers, which may be an intermediate pathway by which maternal obesity affects child health. In addition, for biotin metabolism, which is known to be impaired by severe obesity,^62^ many phage genes are also observed to be enriched in infants from mothers with BMI below 25 in our data. The mothers enrolled in the cohort participated in a randomized clinical trial where they were randomized to receiving fish oil or a placebo from week 24 of pregnancy to 1 week after birth.^42,63^ The design also examined the difference in vitamin D between high and standard doses,^43^ which had no effect on the viral community in our analysis. Of note, the supplementation of fish oil during pregnancy was not found to influence the gut bacterial component at age 1 year. Here, we report that the same intervention has some influence on the gut virome at age 1 year, but the effect is only borderline significant. The infants of mothers that received fish oil had viral genes involved in lysine biosynthesis, glycerophospholipid metabolism, and purine metabolism – metabolic activities that have been associated to fish oil supplementation,^64,65^ but never attributed to gut virome composition. Interestingly, most of these metabolism-related genes were conserved across temperate vOTUs targeting *Ruminococcus*, *Faecalibacterium* and *Prevotella* spp. (Figure 4(b)). These genera have been consistently reported to be enriched in Danish and American subjects with a diet rich in carbohydrates, resistant starch, and fibers, and being determinants of the so-called *Prevotella*-enterotype.^66,67^ The *Prevotella*-enterotype is established early in life (between 9 and 36 months of age)^68–70^ and have been previously suggested as markers of GM maturity at age 1 year.^2,71,72^ Stokholm et al. (2018) reported delayed GM maturation as a risk factor for later development of asthma indicating the importance of these microbes for immune maturation.

Although our study is currently unable to assess how these gut virome-associated genes are actively involved in either enhancing either phage or host fitness, or both, our data as well as our recent finding, that early life gut virome imbalance is associated with increased asthma before school age risk^22^ underlines the potential importance of bacteriophage-encoded metabolic genes and delivers an initial insight of the type of metabolic content conveyed by the gut virome in association to environmental variables.

In summary, our data provide detailed insight into the influence of common environmental factors that shape the gut virome during early life. We also uncover that key gut metabolic functions can be encoded by viral genes, which suggest that, in addition of shaping gut bacteriome composition, phages may directly play a role in metabolic activities.

## Methods

### Study participants

Participants belong to the COPSAC2010 cohort.^34^ Fecal samples for virome extraction sequencing and analysis were collected from all infants at age 1 year.

### Sample collection, sequencing, virome assembly

Preparation of fecal samples, and extraction and sequencing of virions was carried out using a previously described protocol.^37^ Briefly, viral-associated DNA was subjected to short MDA amplification and libraries were prepared following using manufacturer’s procedures for the Nextera XT kit (FC-131-1096 Ilumina, California). Libraries were single-end high-throughput sequenced on the Illumina HiSeq X platform. Details of the pipeline for data processing, de-novo assembly, quality control, bacterial-host and lifestyle predictions, abundance-mapping (vOTU table), and taxonomy of complete and partial viral genomes (here termed vOTUs) can be found in Shah et al.^9^ 16S rRNA gene amplicon data (bOTU table) from the same cohort’s participants were retrieved from Stokholm et al. (2018).

### Environmental exposures

Briefly, during scheduled visits to the COPSAC clinic, information on a wide range of exposures was collected. We included a subset of variables, which were typical host, social and environmental factors suspected to influence the early life microbiome/virome. To avoid potential redundancy, we applied vifcor (Variance Inflation Factor and correlation-based filtering) in the R package “usdm” to identify and remove collinear variables, leading to the current variable list. A total of 30 environmental exposures were investigated and were grouped into social (*n* = 6), pre- (*n* = 4), peri- (*n* = 9) and postnatal (*n* = 11) exposures based on whether they occurred or existed before birth. See Supplementary Table S1 for a complete list of the exposures. For numeric variables like birth weight, we wanted the variable to capture being born with a low birth weight, and in pediatrics, this is defined by a weight <2.5 kg. These classifications of living areas are based on an objective measure of the living environment. This living environment variable included the land cover in a 3 km radius around the home address at birth using the European CORINE satellite-based land cover database.^73^

### Statistics and data analysis

Analyses on diversity were carried out on contingency tables gathering vOTUs abundance. Abundance data was normalized by reads per kilobase per million (RPKM). Alpha-diversity (Observed vOTUs and Shannon Index) indices and Beta-diversity (Bray-Curtis and Sørensen-Dice distances) matrices were generated using the package phyloseq (version 1.42.0).^74^ The contribution of each covariate to explain vOTUs community structure (as determined by Sørensen-Dice similarity and Bray-Curtis dissimilarity metrics) was calculated using distance-based redundancy analysis (db-RDA) models coupled to ANOVA-like permutation test (n permutations = 999) in package vegan (version 2.6–2),^75^ while the effect size of the same covariates on alpha-diversity was calculated with linear mixed models from the package lmerTest (version 3.1–3).^76^ All linear mixed models accounted for technical variation between runs using sequencing lane as the random effect. None of the exposures exhibited significant differences in dispersion.

Different differential abundance analysis methods were evaluated by DAtest.^77^ DESeq2 (version 1.36.0) performed well with a low false-positive rate and a high ability to detect differential vOTUs for our data.^78^ The sequencing lane was considered as a factor-covariate. The raw reads count table of each sample for vOTUs were prepared as input. All parameters are default except for sfType which is set to poscount. The Benjamin-Hochberg method was adapted to correct the p-values. vOTUs with adjusted p-value ≤0.001 and log_2_ fold change ≥ |1| were selected for downstream analyses.

Spearman’s rank correlations were used to test univariate associations of continuous data, and results were visualized in a heatmap. MAFFT^79^ was used to generate the phylogenetic tree file for those highly correlated vOTUs. The phylogenetic tree was visualized using the R package ggtree (version 3.4.0).^80^ Procrustes analysis (R package vegan) was performed on vOTUs as target block and 16S rRNA gene data as rotatory block (n permutations = 999), while using the first two constrained components (CAP1 and CAP2) of db-RDA models for each data block. Bacterial and viral communities were each ordinated via principal coordinates analysis (PCoA) or db-RDA, and their unconstrained coordinate axes were used to represent overall community composition. The “mediation” R package was applied to assess whether bacterial composition partially mediates the early-life exposure effects on viral composition.

ORF calling on selected vOTUs was executed with Prodigal.^81^ To determine metabolic function, genes were annotated based on KEGG Orthology using KofamScan^45^ and filtered by default thresholds. Enricher function in clusterProfiler package (version 4.6.0) was applied to detect whether genes in differently abundant vOTUs were enriched in the metabolic pathway.^82^

All analyses were carried out in R (version 4.0.2) and results were visualized with the package ggplot2 (version 3.3.6).^83^

## Supplementary Material

supplement.docx

## Data Availability

Sequencing FASTQ files are available on ENA under project number PRJEB46943. All cohort participants’ individual-level data are protected by Danish and European law and are not publicly available. However, participant-level data can be made available under a data transfer agreement as part of a collaboration effort. Codes for data analyses are available on GitHub (https://github.com/Yichang07/EarlyVir.git).
