# Supplementary material for: The influence of early life exposures on the infant gut virome
Source: Gut Microbes. 2025 May 21;17(1):2501194. doi: 10.1080/19490976.2025.2501194 (PMC12101590; doi:10.1080/19490976.2025.2501194)
Supplement: supplement.docx [file KGMI_A_2501194_SM1964.docx]

**
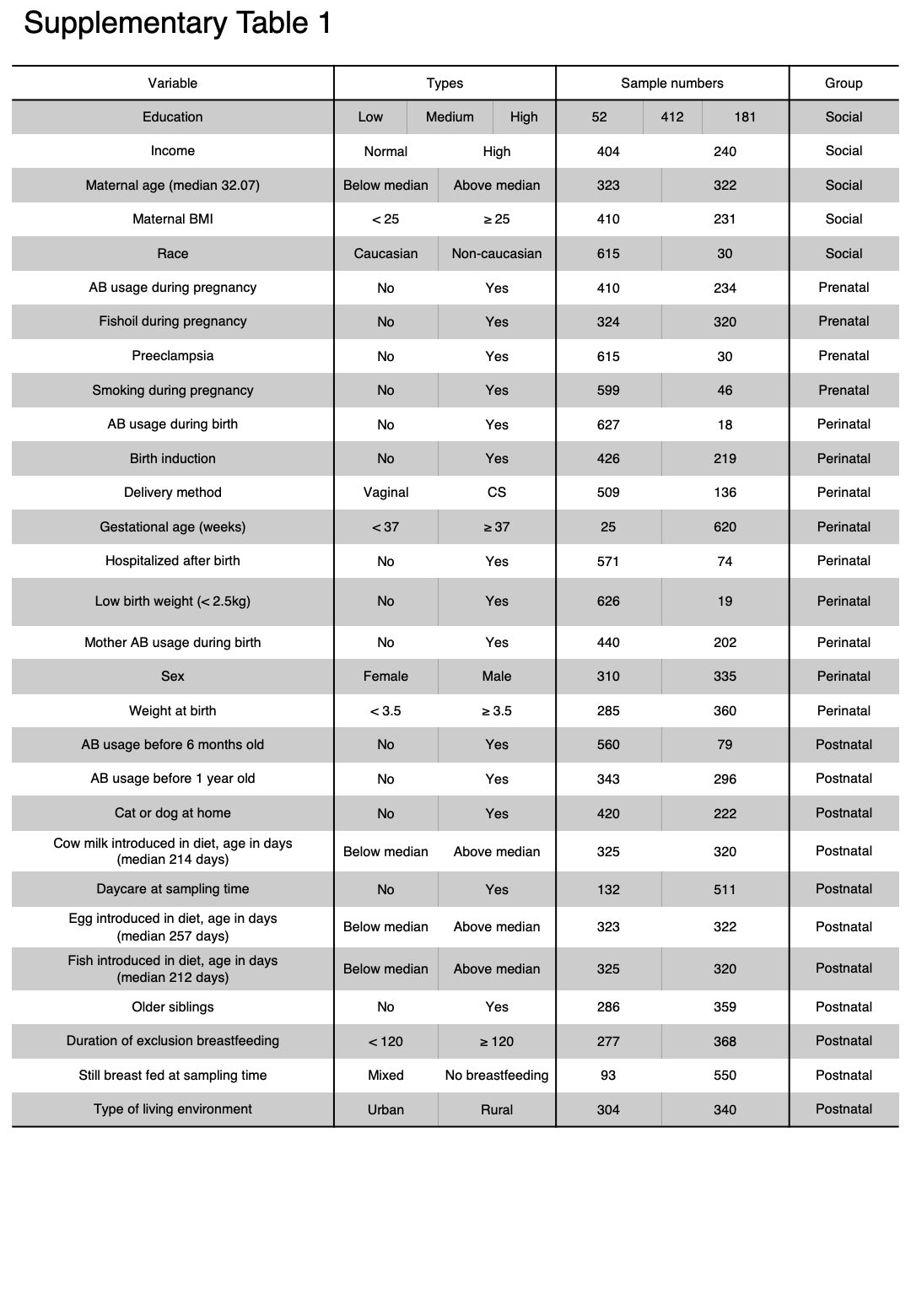
**

**Supplementary Table 1. Environmental exposure characteristics of the infant samples**

Column “types” shows the composition of each exposure. Column “numbers” depicts the number of samples (N) for each factor. The group column shows how each variable is grouped.


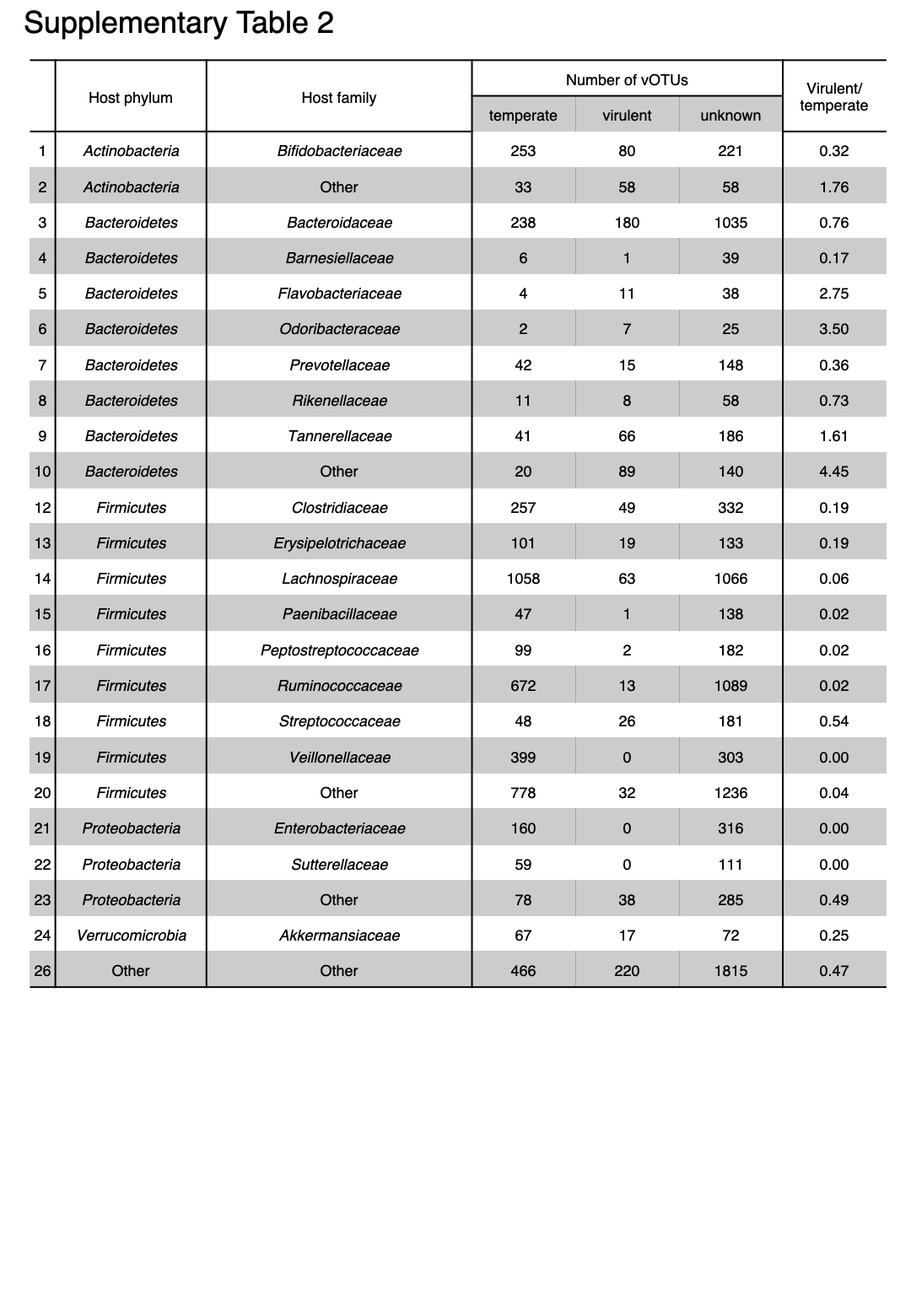


**Supplementary Table 2. Number of different lifestyle vOTUs**


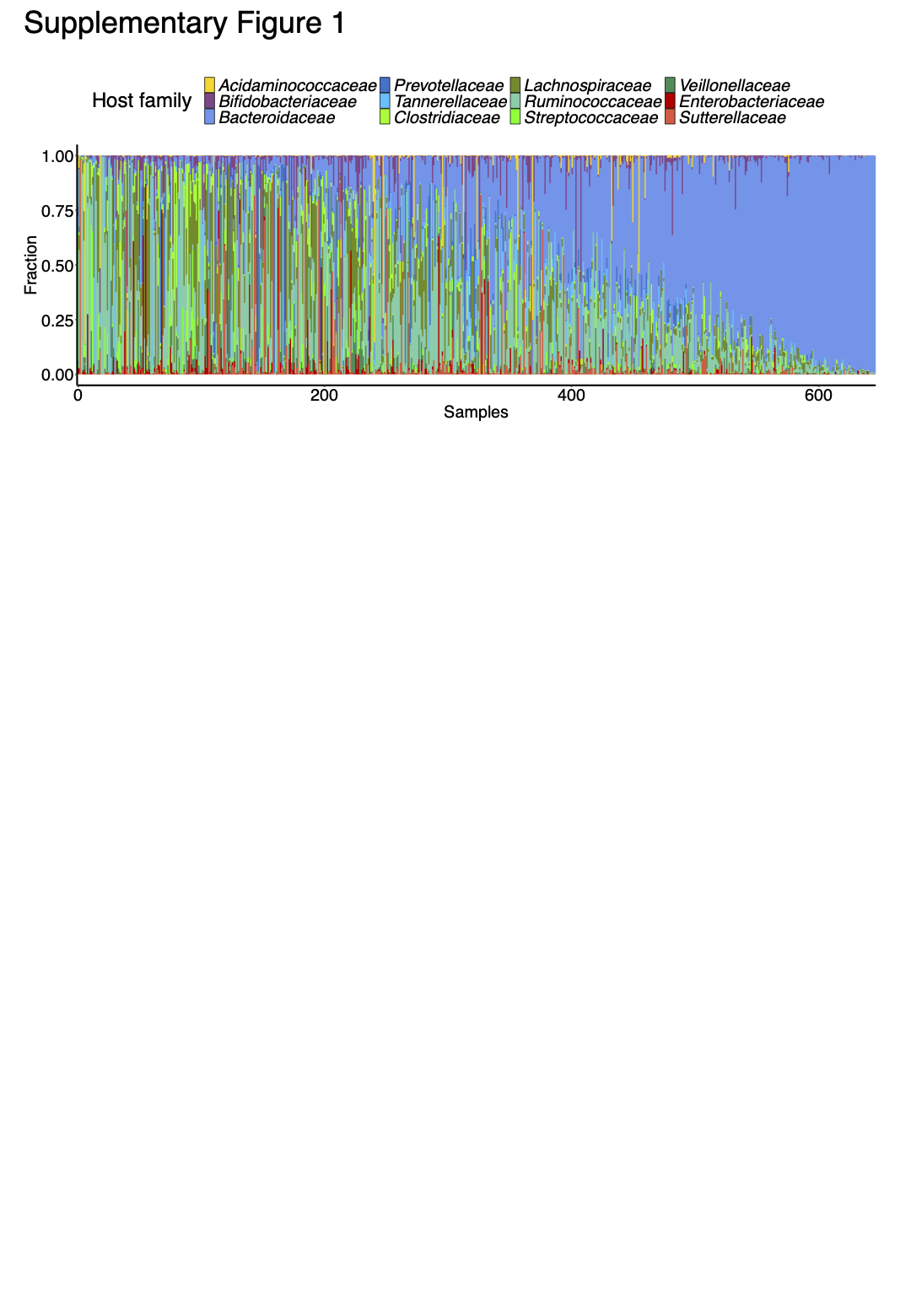


**Supplementary Figure 1. Relative abundance of vOTUs across all samples grouped by their host bacterial family**

**Supplementary Figure 2. Additional beta diversity analysis of environmental factors on gut virome**

(A-B) The effect size of exposures on virome variation calculated by db-RDA on Bray-Curtis (A) and Sorensen-Dice (B) dissimilarity matrices.

(C-D) Barplot showing the strength of associations (-log_10_ adjusted p values) of environmental exposures with viral composition using db-RDA based on Bray-Curtis dissimilarity (C) and Sorensen-Dice distance (D) matrices. P-values were adjusted by Benjamini–Hochberg method. The red line is the q = 0.05 cut-off and the black line is the q = 0.2 cut-off.


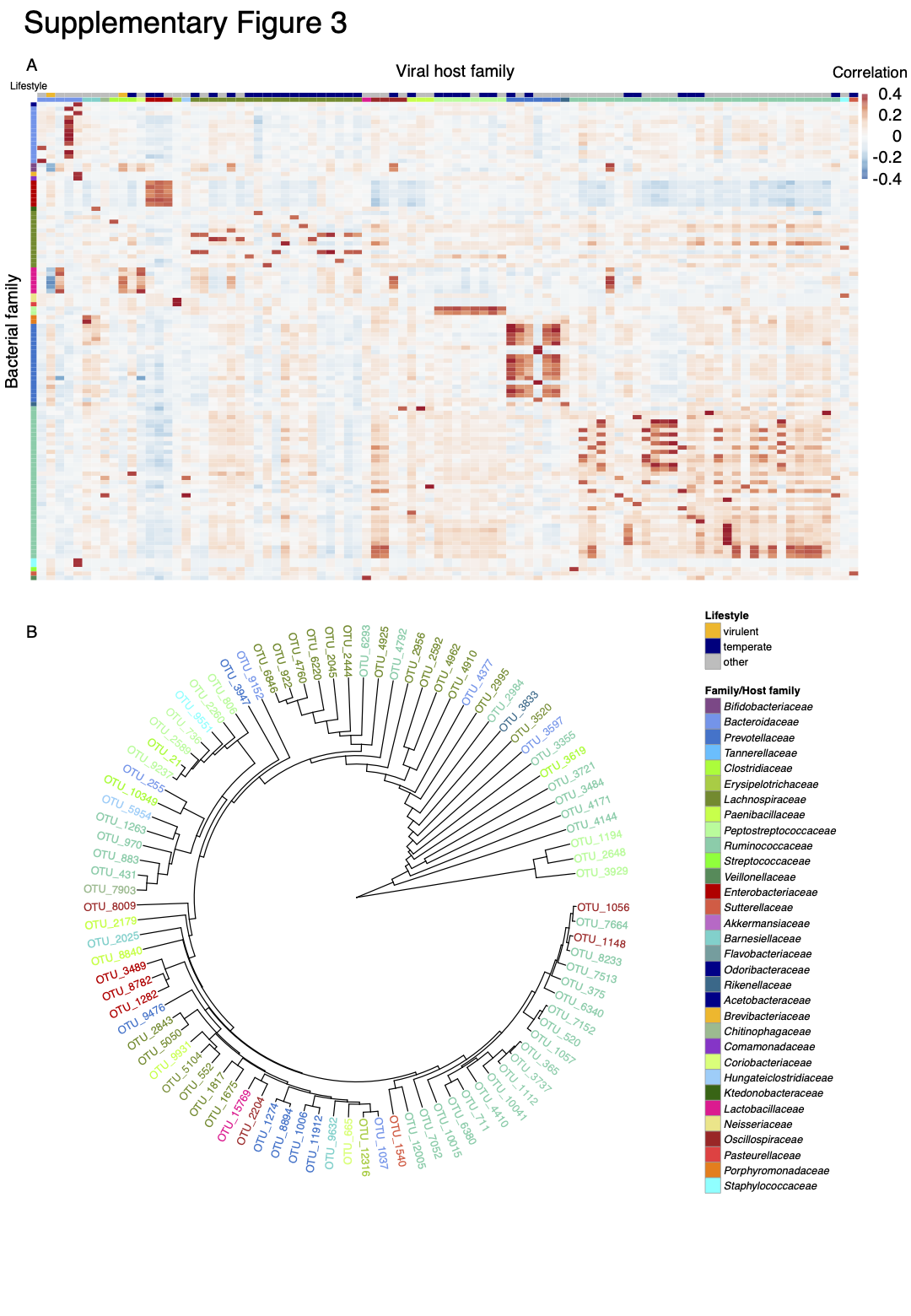


**Supplementary Figure 3. Co-abundance of vOTUs and bacterial OTUs and the phylogeny of vOTUs**

(A) Heatmap of Spearman’s rank correlation between the differentially abundant vOTUs and 16S rRNA (V4 region) data blocks. The rows represent the 91 vOTUs, and the columns represent the 110 bacterial OTUs. Virome contigs were labeled according to their host family. Bacterial OTUs were labeled according to their family.

(B) Phylogenetic tree showing genetic relationships of vOTUs.


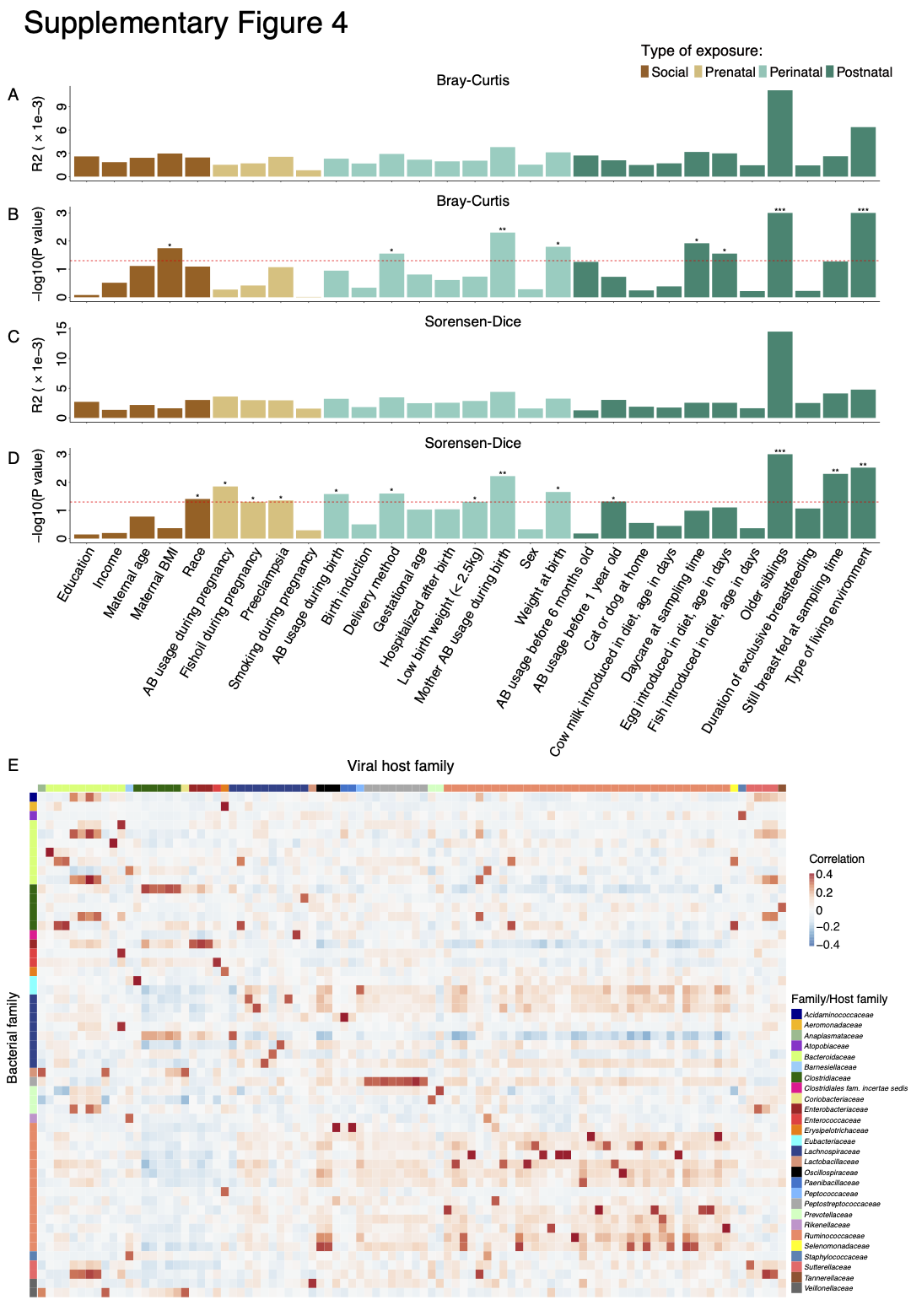


**Supplementary Figure 4. Beta diversity analysis of environmental factors on gut bacterial community and additional co-abundance analysis**

(A-D) Barplots showing p-values and effect sizes of db-RDA analysis for bacterial variation based on Bray-Curtis (A-B) and Sorensen-Dice (C-D) dissimilarity matrices. P-values were calculated by an ANOVA-like permutation test (n = 999).

(E) Heatmap of Spearman’s rank correlation between the differentially abundant vOTUs and whole-genome shotgun metagenome data blocks.

**Supplementary Figure 5. Mediation analysis of early-life exposures, bacterial community composition, and viral community**

(A-D) Estimates from a causal mediation analysis for quantifying the bacterial contribution (Indirect effect) to the exposure effect on viral community (Direct effect) are shown in forest plots. Dots indicate point estimates and horizontal lines indicate 95% CIs.


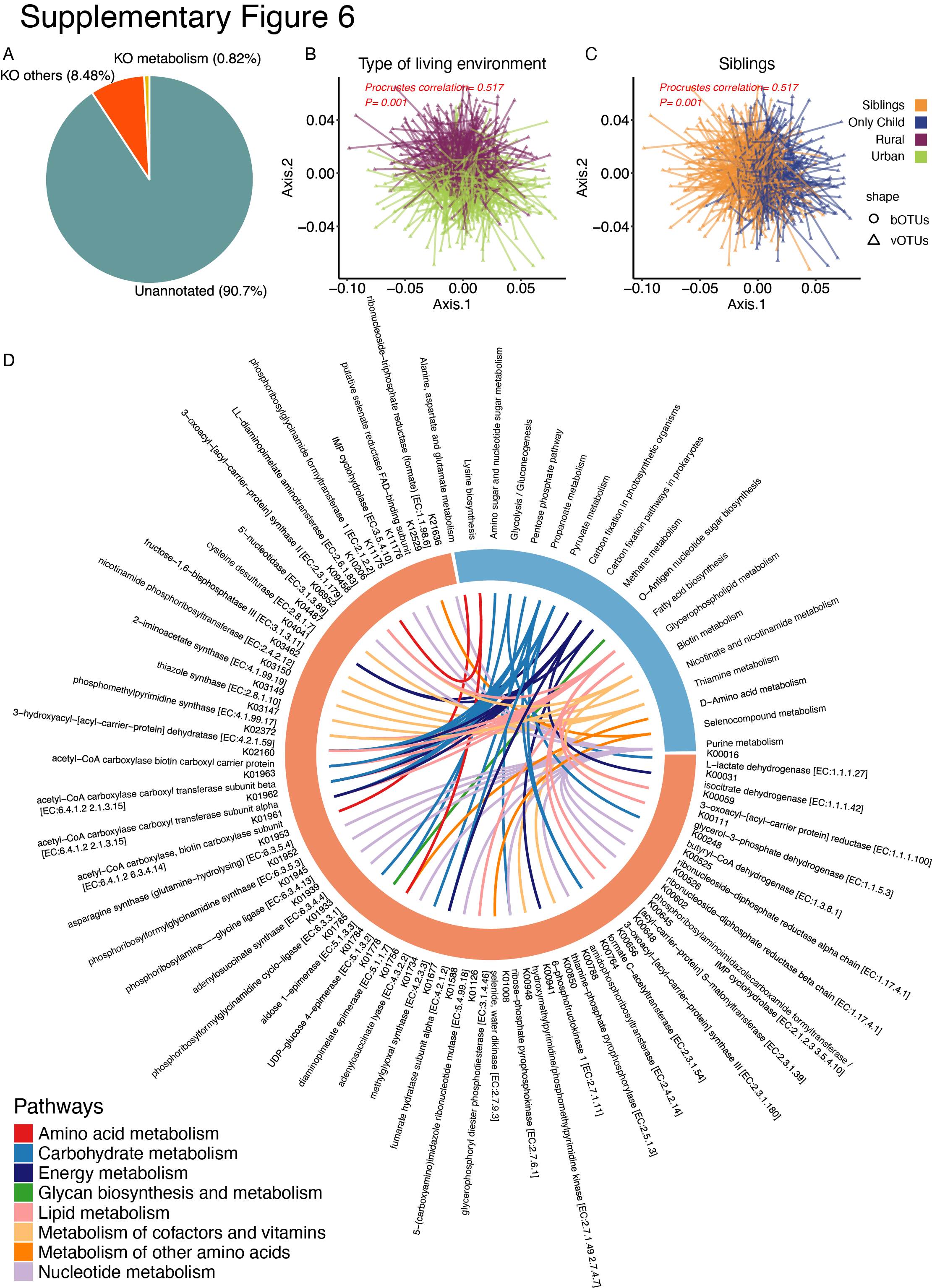


**Supplementary Figure 6. The potential of phage-host association and enzymes encoded by phages**

(A) Distribution of gene annotations to KEGG databases.

(B-C) Procrustes correlation between the virome data and bacterial 16S rRNA gene data. Sorensen-Dice was used to generate distance matrices, the results of db-RDA were taken into the procrustes analysis. The triangle represents the virome samples and the circle represents the bacteria samples.

(D) KOs (enzymes and E.C. numbers) that are associated with metabolism pathways.
